# Gold Nanoparticle-Based Detection of Low Molecular Weight AGEs from In Vitro Glycated Haemoglobin A0 Samples

**DOI:** 10.1186/s11671-018-2812-y

**Published:** 2018-12-04

**Authors:** A. Asha Madhavan, S. Juneja, P. Sen, R. Ghosh Moulick, J. Bhattacharya

**Affiliations:** 1 School of Biotechnology, Jawaharlal Nehru University, New Mehrauli Road, New Delhi, 110067 India; 2 Amity Institute of Integrative sciences and Health, Amity University Gurgaon, Manesar, Haryana 122413 India; 3 School of Physical Sciences, Jawaharlal Nehru University, New Mehrauli Road, New Delhi, 110067 India

**Keywords:** In vitro glycation, Advanced glycation end products, Haemoglobin A0, Colloidal gold nanoparticles, Surface plasmon resonance, Colorimetric sensor

## Abstract

Protein glycation is a major biochemical event that takes place in the plasma of diabetic patients due to increased sugar levels. Extensive glycation leads to the formation of advanced glycation end products (AGEs) that is well known for having detrimental effects on diabetic patients. In the current work, we have glycated the physiologically important protein Haemoglobin A0 in vitro to study AGE formation and activity by using them as a template for gold nanoparticle (GNPs) synthesis. It was found that the surface plasmon resonance of synthesised GNPs showed high correlation with the extent of glycation. On fractionation, the glycated Haemoglobin A0 segregated into two distinct population of products, one consisting of proteinaceous, cross-linked larger fragments of Haemoglobin A0 and a second population of non-proteinaceous low molecular weight AGEs. Only low molecular weight AGEs contributed to synthesis of GNPs upon using the fractions as a template, substantiating the principle of proposed GNP-based assay. Owing to its physiological importance, AGEs can be used as a diagnostic means for diabetes and its associated complications. In this study, we have employed the high reactivity of AGEs for the development of a GNP-based novel colorimetric sensor to enable their detection. Our proposed GNP-based sensing could have high clinical significance in detecting diabetes and its associated complexities.

**Electronic supplementary material:**

The online version of this article (10.1186/s11671-018-2812-y) contains supplementary material, which is available to authorized users.

## Background

Type II diabetes mellitus (T2DM) is a complex metabolic disorder characterised by the presence of high blood sugar levels. The hyperglycaemia persisting in the body initiates a series of chemical and biochemical reactions and the most important reaction that occurs during the course of hyperglycaemia is known as Maillard reaction which involves non-enzymatic reaction between sugars and protein to form a reversible aldimine (Schiff base) linkage. The relatively unstable Schiff base tautomerises into its more stable keto form which is otherwise known as the ‘Amadori Product’ [1]. Amadori products, also referred to as intermediate products of glycation or early glycation products, undergo group rearrangements, cyclisation, dehydration, degradation reactions to form a range of chemical compounds. These compounds are known to be potential cross linkers of protein and glycosylating agents [2]. They are susceptible to further degradation leading to the formation of advanced glycation end products (AGEs) [3]. At high endogenous concentrations, AGEs contribute to the complications of diabetes [4] either by inducing protein crosslinks or through receptor mediated intracellular signal transduction (RAGE), leading to oxidative stress and inflammation [5, 6]. It is also known to be associated with cardiovascular diseases [7, 8], nephropathies [9, 10], retinopathies [11, 12], ageing [13, 14], arthritis, cancer [15–17], neurodegenerative diseases like Alzheimer’s [15] and development of depression [18]. Besides Maillard reaction, several other pathways including oxidation of glucose, lipid per-oxidation and polyol pathway also leads to the formation of AGEs [19, 20] in vivo. In addition to that, dietary intake of certain food items which are known to be rich in AGEs also contribute to the total AGEs inside the body [6].

Although most of the AGE products have a common structural element [21], the exact chemical structures of hundreds of AGEs are yet to be identified [22]. Despite their heterogeneity, formation of covalent cross links with the protein and the ‘browning effect’ are typical for all the known AGEs [23]. The adducts involving sugar and protein which are formed at the early stage of glycation are referred to as early glycation adducts. Fructosyllysine and Fructosamine are examples of such structures and they are potential cross-linkers [24–26]. Some of the widely studied AGEs include *N*-carboxymethyl-lysine (CML), *N*-carboxyethyl-lysine (CEL) [27], pentosidine [28], glucosepane [29], pyrraline, Argpyramidine, Crossline and Vesperlysine C [30].

Studies on the role of AGEs in the progression of diabetes, ageing and associated complications are on a rise and they have been reported to be a good biomarker for the respective studies [https://clinicaltrials.gov/ct2/show/NCT02863224][31–34]. However, the complexity and heterogeneity in the structure of AGEs is a major hurdle in the development of a universal detection system [35]. Qualitative analysis of AGEs is generally done through spectroscopic and colorimetric techniques [36] in which the evolution of Maillard reaction products is monitored by either measuring the enhancement of light absorption at 280 nm which corresponds to the early Maillard reaction products [37] or by measuring the fluorescence emission at 450 nm [38–42]. Studies for the identification of distinct AGE products are still underway; however, some attempts were made for the quantitation of pentosidine [43] and carboxymethyl lysine [44]. Most of the AGEs were studied on the tissue level by immunohistochemistry and ELISA-based quantification using a range of poly- and monoclonal antibodies [21, 45]. The best analytical technique available to date for AGE detection is liquid or gas chromatography followed by spectrophotometric or mass spectrometric detection [12, 46–49]. The quantitative analysis of AGEs is still a major technical challenge and the few techniques available for the same are expensive and thus limits its use in point-of-care applications. Development of novel strategies for the qualitative and quantitative evaluation of AGEs is the need of the hour owing to their implications in life threatening diseases.

Great efforts have been put to the development of colorimetric sensors owing to their simplicity in detection, fast response time and cost effectiveness especially for biological applications [50]. Among them, surface plasmon resonance (SPR)-based sensors are of particular interest due to their high sensitivity of detection [51]. In the present study, we have explored the optical properties of gold nanoparticles (GNPs) for the qualitative identification of AGE products. GNPs are known for their unique, tuneable SPR and therefore evolved as a colorimetric reporter for the detection of various biomolecules. Optical sensors based on GNPs include those for small chemical analytes [52], sugars [53], different proteins [54], protein aggregates [55] and conformational variants of a protein [56]. Previously, we showed that GNPs when seeded on a glycated protein template could respond to the advancement of glycation [57]. Here, we have extended this concept for the qualitative detection of different glycation products colorimetrically by employing the reducing property exhibited by the AGEs. Briefly, HbA0 was glycated in vitro, using fructose as the reducing sugar, AGE formation and the associated structural changes in the protein backbone was confirmed using spectroscopy. The glycated Hb was fractionated using gel filtration chromatography to separate the products based on their molecular weight and GNPs were synthesised using the fractions obtained. Only the non-proteinaceous AGE products directed the synthesis of gold nanostructures thus enabling their identification.

Growing evidences support the involvement of different products of glycation in diseases including diabetes, ageing, Alzheimer’s, renal diseases, atherosclerosis and different types of cancers. Our findings accentuate the use of GNPs as a simple and highly sensitive colorimetric sensing platform for the identification of different AGE products which could be implicated in the prognosis of diabetes associated health complications.

## Methods

### Materials

Haemoglobin A0 (HbA0) and Sephadex (G25) were obtained from Sigma Aldrich India Pvt. Ltd. Hydrogen tetrachloroaurate (III) trihydrate (HAuCl_4_.3H_2_O) was purchased from Loba Chemie. Fructose, potassium dihydrogen orthophosphate, dipotassium hydrogen orthophosphate, sodium dihydrogen orthophosphate, disodium hydrogen orthophosphate, potassium ferricyanide, trichloroacetic acid, ferric chloride, nitric acid (HNO_3_), hydrochloric acid (HCl), acrylamide, bis acrylamide, ammonium persulfate, TEMED, sodium dodecyl sulphate, Coomassie Brilliant Blue, glycerol, dithiothreitol, TRIS base and other chemicals used were of analytical grade and used without further purification. MilliQ ultrapure water (>18 MΩ) was used for all the experiments.

### Methods

#### Glycation of HbA0

Stock solutions of HbA0 and fructose were prepared in autoclaved potassium phosphate buffer (0.1 M, pH 7.4) and filtered using 0.2 μm syringe filters prior to use. HbA0 samples were incubated with different concentrations of fructose for different time periods (1 to 10 days) in an incubator at 37 °C. The final concentrations of fructose and HbA0 were 0.1 M and 1 mg mL^−1^ respectively. Controls of HbA0 and fructose were also kept which were of the same concentrations. The samples which were glycated for 10 days are labelled with a prefix of ‘Day 10’ and the control samples which were not incubated are labelled with the prefix ‘Day 0’. All the experiments were performed under sterile conditions in a laminar air flow hood.

#### Reducing Property Assay

The reducing properties of the glycated samples were determined by the method described by Gu. et al. [37] with minor modifications. Then, 100 μL of the glycated samples and their respective protein and sugar controls were mixed with 1 mL of 0.2 M sodium phosphate buffer and 1 mL 1% potassium ferricyanide. The mixtures were incubated at 50 °C in a water bath for 20 min. After cooling the mixture to room temperature, 1 mL of 10% trichloroacetic acid was added. To 1 mL of this mixture, 1 mL of MilliQ water and 200 μL of 0.1% ferric chloride were added. The absorbance of the resultant mixture was measured at 700 nm. A negative control was kept in which 0.1 M phosphate buffer was added in place of the glycosylated sample and this served as a blank for absorbance measurements. The higher the absorbance at 700 nm, the more its reducing property.

#### Gel filtration Chromatography of Day 0 Fruc-Hb and Day 10 Fruc-Hb Using Sephadex (G25)

Briefly, Sephadex (G25) beads were soaked in MilliQ water overnight, then packed in a glass column of 15 cm length, 1 cm diameter and 15 mL inner volume. At first, the column was washed with copious amount of MilliQ water followed by potassium phosphate buffer (0.1 M, pH 7.4). Approximately 600 μL of the Fruc-Hb sample was loaded into the column after equilibrating the same with phosphate buffer. Elution was carried out with the same buffer at a flow rate of 7.5 mL per hour. Fractions were collected once the loaded sample crossed approximately 10 cm distance of the column. Approximately 30 fractions were collected from each of the samples and were characterised thereafter. Here, day 0 Fruc-Hb and the fractions derived from it served as a control against the day 10 Fruc-Hb sample and their respective fractions.

#### Gold Nanoparticle Synthesis

All the glasswares used for GNP synthesis were washed with Aqua Regia (HCl:HNO_3_ in a 3:1 ratio by volume) and rinsed with ethanol and ultrapure water prior to use (*Caution! Aqua Regia is a very corrosive oxidising agent which should be handled with great care*). The synthesis of GNPs were carried out, exploiting the reducing property of glycation products of Hb. In a typical experiment performed at room temperature (RT), 32 μL of aqueous solution of HAuCl_4_ (1% *w*/*v*) was added to 3.868 mL of MilliQ water under constant stirring. Upon complete dissolution of the gold salt, the solution turns pale yellow. Next, 50 μL of the requisite Fruc-Hb sample or 100 μL of the fraction was added to the gold salt solution. Once all the reactants were thoroughly mixed, stirring was stopped and the reaction mixture was left undisturbed to allow the growth of GNPs. In the final reaction mixture, the concentration of Fruc-Hb sample was 12.5 ng μL^−1^ (12.5 μg mL^−1^) and that of the fraction was 1 ng μL^−1^ (1 μg mL^−1^) (The detailed calculations are given in S6). Colours ranging from pink to purple developed in the reaction mixtures depending on the sample added. All the samples were maintained till the colour of the reaction mixture stabilised and no further change was observed.

#### Bradford Assay

The fractions collected from Fruc-Hb samples were analysed for the presence or absence of protein using Bradford assay. Briefly, to each of 20 μL of undiluted fractions, about 200 μL of Bradford reagent was added, and the absorbance was measured at 595 nm and 450 nm [58]. The ratio of OD at 595 nm to 450 nm was plotted against fraction number and a higher ratio indicated relatively higher percentage of the protein.

### Spectroscopy

#### UV-Visible Spectroscopy

UV-Visible spectra of all the Fruc-Hb samples, their respective controls and the chromatographic fractions from the glycated samples were recorded in a Perkin Elmer, Lambda 25 UV-Visible spectrometer. Spectra were taken by running a scan from 800 to 200 nm with a scan speed of 240 nm/min, and slit width of 1 nm. All the measurements were taken using 1 cm path length, 1 mL Quartz cuvettes. The UV-Visible spectra of GNPs were also recorded in a similar manner.

#### Fluorescence Spectroscopy

To identify protein structural alterations and AGE formation, the fluorescence emission profiles of Fruc-Hb samples and the fractions from day 0 Fruc-Hb and day 10 Fruc-Hb were performed using Agilent Technologies Cary Eclipse fluorescence spectrophotometer. Excitation and emission slit widths were set to 5 nm and spectra were taken using a quartz cuvette of 1 cm path length. Samples were excited at 280 nm and 350 nm for checking the tryptophan fluorescence/intrinsic protein fluorescence and AGE fluorescence respectively [34–38].

#### Circular Dichroism Spectroscopy

The secondary structure alterations in the Fruc-Hb samples were identified using circular dichroism spectroscopy. The measurements were performed using circular dichroism (CD) spectrometer with Stop Flow, Applied PhotoPhysics Chirascan (Applied Photophysics Limited, UK). The spectra were taken in the far UV, ranging from 190 to 260 nm.

For all the spectroscopy measurements, Fruc-Hb samples of 0.1 mg mL^−1^ concentration were used and fractions from day 0 Fruc-Hb and day 10 Fruc-Hb were analysed without any dilution. Potassium phosphate buffer (pH 7.4, 10 mM) served as a blank in all the experiments. All the readings were taken in triplicates at room temperature.

#### Transmission Electron Microscopy

To identify the size and structure of the GNPs synthesised from the Fruc-Hb samples and their respective controls, electron microscopy analysis were performed using transmission electron microscope (TEM)–JEOL 2100F. The GNPs were concentrated by centrifugation at 6000 rpm and were drop casted onto copper-coated carbon grids of mesh size 300. The samples were allowed to dry overnight at room temperature prior to the analysis.

#### Colour Intensity Profile of GNPs

In order to express the colour of GNPs in measurable values, (R-B)/G ((Red intensity–Blue Intensity)/Green intensity) was calculated by extracting red, blue and green colour planes from each of the gold colloids using a digital colour meter.

#### SDS Polyacrylamide Gel Electrophoresis

Sodium dodecyl sulphate-polyacrylamide gel electrophoresis (SDS PAGE) was performed in order to see the differences in the molecular weight of HbA0, post glycation. HbA0 Controls of day 0, day 10 and Fruc-Hb of day 0 and day 10 were mixed with 10% SDS containing 6X loading buffer and boiled for 5 min following which 30 μL of the samples were loaded onto the casted gel (Stacking gel-5%; Resolving gel − 12%). The samples were run along with the 10-175 kDa PiNK Plus pre-stained protein ladder (Cat No. PM005 0500) at 100 V using Mini PROTEAN Tetrad system from Bio-Rad. The gels were visualised under the white light source of a Bio-Rad Trans illuminator.

## Results

### GNP Synthesis from Fructose, Haemoglobin A0 and Glycosylated Haemoglobin A0

**Fig. 1:**
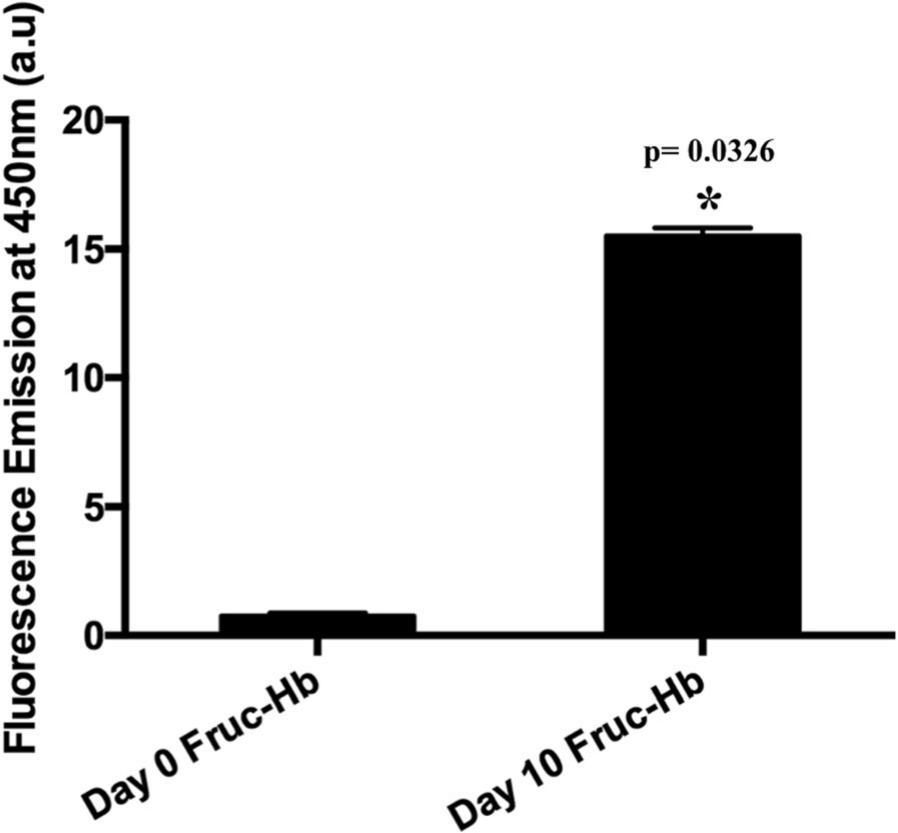
Formation of AGEs measured by generation of fluorescence at 450 nm when haemoglobin is incubated with fructose for 10 days. Comparison of 450 nm fluorescence emission in day 0 Fruc-Hb and day 10 Fruc-Hb

It is known that the reducing sugar glucose react with HbA0 in vivo to produce HbA1c, the well-known early glycation adduct which is a major biomarker for the hyperglycaemic levels associated with diabetes [59]. To achieve faster glycation kinetics and high AGE accumulation in vitro, fructose is used in place of glucose which is a potent glycating agent compared to the latter [55–60]. In our study, we performed the glycation of Haemoglobin A0 using fructose as the reducing sugar and the glycation was monitored till 10 days which is reported to be good enough for substantial AGE formation [57]. Figure 1 illustrates the formation of AGEs in the HbA0 samples after 10 days of incubation with fructose in vitro. AGE formation was evaluated by qualitatively measuring the fluorescence emission at 450 nm upon excitation at 350 nm [38–42]. More than tenfold increase in fluorescence emission at 450 nm confirmed formation of AGE products in glycosylated HbA0 (day 10 Fruc-Hb) compared to the non-glycosylated HbA0 (day 0 Fruc-Hb- Physical mixture of HbA0 and fructose without incubation). The detailed biophysical characterisation of the samples is discussed in the Additional file 1 (S1 & S2).

**Fig. 2:**
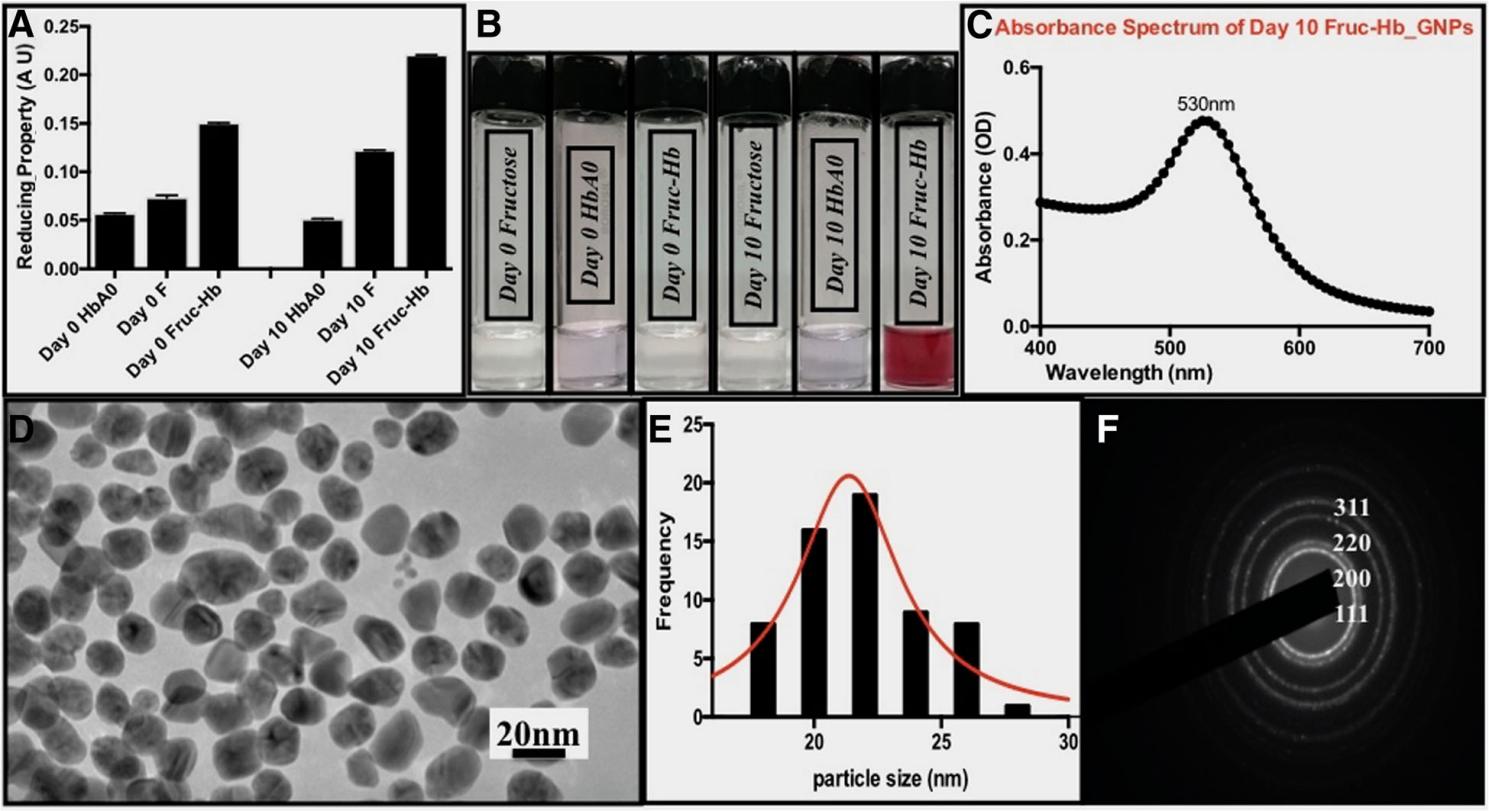
Characterisation of AGE formation and GNP synthesis from HbA0, fructose and Fruc-Hb samples. **a** Reducing properties of HbA0, fructose, Fruc-Hb of 0 and 10 days of incubation respectively measured by the ferric ion reduction test. **b** Photographs of GNPs synthesised from the respective samples. **c** UV-Visible absorption spectrum for the day10 Fruc-Hb_GNPs. **d** Transmission electron micrograph of the day10 Fruc-Hb_GNPs (scale bar: 20 nm). **e** Size distribution of the particles and **f** SAED for the particles

In order to use GNPs as a colorimetric sensor for AGEs derived from protein glycation, it was a prerequisite to study the formation kinetics of GNPs from the sugar and protein reactants. Fructose and Haemoglobin A0 are already reported to be directing the synthesis of gold nanostructures [53, 61–63] when used alone or in combination with a strong reducing agent. However, to understand the difference in formation kinetics, we compared the GNPs synthesised from Fruc-Hb, fructose and HbA0 incubated for 0 and 10 days respectively in the absence of an additional reducing agent. The reducing property of these templates were measured biochemically according to the protocol described in the methods section. Overall, the day 10 samples showed higher reducing property than the day 0 samples, and Fruc-Hb exhibited the maximum reducing property followed by fructose and HbA0 in both the cases, which could be attributed to the presence of AGEs in Fruc-Hb samples (Fig. 2a). Out of these samples, at a typical concentration, only day 10 Fruc-Hb contributed to the synthesis of stable GNPs (Fig. 2b) by the end of 4 days, whereas the rest of the samples could do so only when they were allowed to be kept for extended time (typically more than 10 days) and the results are in accordance with the obtained reducing properties of the samples (data not shown). The visible light absorption spectrum of the day 10 Fruc-Hb_GNPs generated a peak around 530 nm (Fig. 2c) and the particles were also characterised by transmission electron microscopy (TEM), to study the size and morphology (Fig. 2d). Electron micrograph showed the presence of spherical particles with an average diameter of 21.930 ± 2.4 nm (Fig. 2e). Also, the particles were polycrystalline in nature with lattice points corresponding to 111, 200, 220, 311 planes of an fcc lattice (Fig. 2f). A detailed kinetic analysis of GNP formation from differentially glycated samples is given in S3.

Here, Fruc-Hb acts both as a template as well as stabiliser to direct the synthesis of gold nanostructures of spherical shape. Since only day 10 Fruc-Hb enables the particle synthesis in a stipulated time compared to the sugar and protein counterparts, this mechanism can be used to differentiate between a glycated and non-glycated protein template.

### Fractionation of Fruc-Hb Resolves Two Distinct Class of Glycosylation Products

**Fig. 3:**
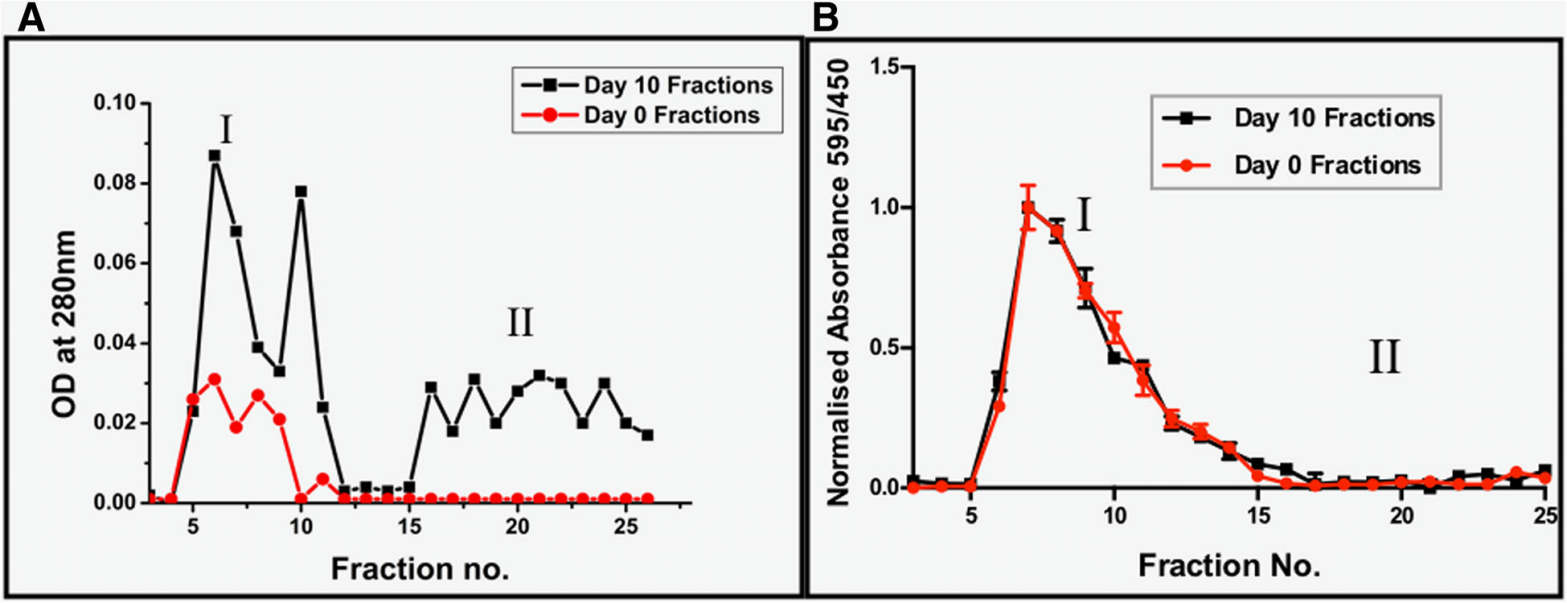
Elution profile of day 0 Fruc-Hb (red) and day 10 Fruc-Hb (black) fractions. UV absorbance profile measured at 280 nm (**a**) and Bradford assay for the presence of proteins (**b**) of fractions from day 0 Fruc-Hb and day 10 Fruc-Hb

It was interesting to see if the AGEs or the alterations in the protein backbone are responsible for the observed differential SPR response in the GNPs seeded on day 10 Fruc-Hb template. To investigate further, we fractionated day 10 Fruc-Hb and day 0 Fruc-Hb using gel filtration chromatography as described in the methods section. The fractions derived from day 0 Fruc-Hb served as a control against the fractions from day 10 Fruc-Hb. Figure 3 summarises the spectroscopic characterisation of the fractions. Figure 3a shows the elution profiles of fractions collected from day 0 Fruc-Hb (red) and day 10 Fruc-Hb (black). The characteristic elution profile obtained for the fractionation of Fruc-Hb is consistent with the earlier reports [37].

The presence of protein in the fractions from day 0 and day 10 was confirmed by the absorption of UV light at 280 nm by the aromatic amino acids present in the protein. The elution profile of day 10 Fruc-Hb measured by light absorption at 280 nm shows the presence of two population of products marked I and II. Population I, consisting of high molecular weight products, ranges from fraction no. 5 to 12 and fraction no. 15 onwards corresponding to low molecular weight products fall in population II. A small range spanning 2–3 fractions was observed in between I and II, where no UV absorption was observed. On the other hand, a single population of fractions from day 0 Fruc-Hb showed UV light absorption (population I), confirming that the absorption at region II in day 10 fractions is by the products generated in day 10 Fruc-Hb as a result of glycation. It is already reported that the intermediates of Maillard reaction absorbs light in the near UV range [64] which supports the observation. The enhanced absorption of UV light at 280 nm by day 10 fractions compared to the day 0 fractions could be due to the extensive unfolding of protein as a result of glycation, resulting in the exposure of aromatic amino acids (Fig. 3a).

Glycation considerably alters the secondary and quaternary structures of the protein as discussed in S1. To confirm the structural status of the protein in the fractions, we performed SDS polyacrylamide gel electrophoresis of the fractions and found that in place of monomeric and dimeric bands, fused bands were observed confirming the cross-linking of protein in day 10 Fruc-Hb sample (Additional file 1: Figure S4).

On comparing the elution profiles of day 0 Fruc-Hb and day 10 Fruc-Hb, we discern that the fractions falling into population I are proteinaceous in nature and the second population which was completely absent in day 0 fractions (non-glycated) consist of non-proteinaceous glycation products. These findings are supported by the protein estimation by Bradford’s assay where the proteinaceous nature of the fractions in population I of both day 0 and day 10 Fruc-Hb was confirmed (Fig. 3b). Concisely, fractionation of day 10 Fruc-Hb by gel filtration chromatography yielded two different populations of fractions, both absorbing light at 280 nm one being proteinaceous in nature and the other being non-proteinaceous.

### The Proteinaceous Glycation Products and Non-Proteinaceous Glycation Products Are Fluorescent in Nature

**Fig. 4:**
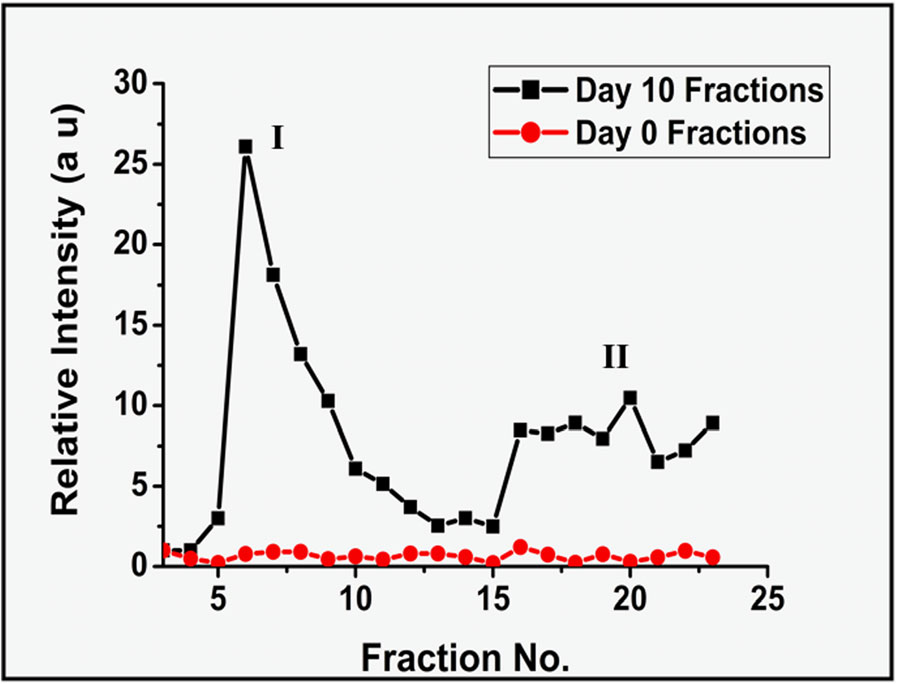
Comparison of AGE fluorescence in day 0 Fruc-Hb and day 10 Fruc-Hb fractions expressed as the intensity of fluorescence emission at 450 nm

Having understood the elution of glycation products of proteinaceous and non-proteinaceous in nature, fluorescence emission of these fractions were measured at 450 nm to characterise the presence of AGE products. Similar to the UV elution profile, two populations of fluorescence emission is observed for day 10 fractions, but none of the day 0 fractions exhibited fluorescence emission at 450 nm since no AGE formation has occurred in day 0 Fruc-Hb (Fig. 4). The intensity of fluorescence varied significantly among the day 10 fractions indicating the differential chemical nature of the glycation products. In the fluorescence elution profile, the initial elutes belonging to population I consisted of glycation products of high fluorescence intensity. This together with the protein estimation shown in Fig. 3 suggests the elution of highly fluorescent glycation products which comprises of proteinaceous structures in these fractions. The second population exhibited fluorescence emission of lesser intensity compared to the initial fractions. They were found to be non-proteinaceous in nature (Fig. 3b) but capable of absorbing UV light at 280 nm (Fig. 3a).

Taking into account the UV absorption (Fig. 3) and fluorescence emission of the day 10 fractions (Fig. 4), it is evident that the fractionation differentiates two different class of glycation products of HbA0. The population of fractions which gets eluted from the G25 column initially are of high molecular weight, proteinaceous in nature and emits strong fluorescence at 450 nm. The second class of products are low molecular weight non-proteinaceous structures, emitting fluorescence at 450 nm but of comparatively lower intensity. Since both products exhibit ‘AGE’ fluorescence, the proteinaceous structures could be the AGE products cross linked to the protein backbone which are formed initially during Maillard reaction and the fractions of the second population could be the late products of glycation which are formed as a result of the degradation of AGE-protein crosslinks.

### GNPs Seeded on Fractions from Fruc-Hb Sample Enables Differentiation Among the Glycation Products

It is evident that synthesis of GNPs can be used to differentiate between a glycated and non-glycated template (Fig. 2). Now, to investigate if the GNPs can differentiate among the proteinaceous and non-proteinaceous elutes from day 10 Fruc-Hb, we synthesised GNPs using the fractions obtained from day 10 Fruc-Hb as described in the methods. The colour of GNPs formed was monitored until all the samples became stable.

**Fig. 5:**
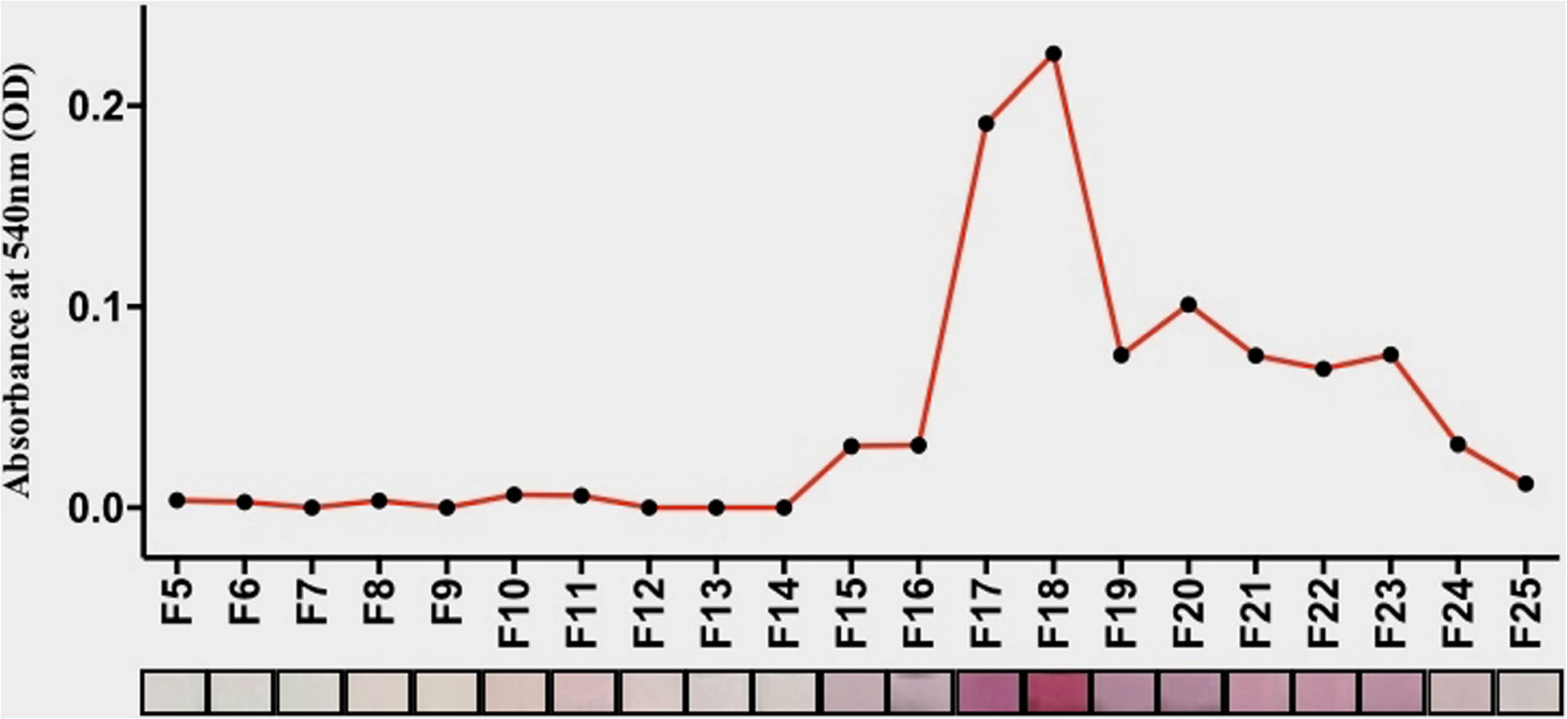
GNP synthesis from day 10 Fruc-Hb fractions. The colorimetric profile of GNPs synthesised from day 10 fractions and their absorption maxima plotted against the fraction number

As shown in Fig. 5, none of the fractions consisting of proteinaceous structures (population I) exhibited notable GNP formation, whereas the fractions containing non-proteinaceous AGE products (population II) produced stable GNPs exhibiting a range of colours in colloidal solution. The formation of GNPs were characterised by using UV-Visible spectroscopy and the absorbance maxima were plotted against the fraction numbers. The spectroscopy data are well in support to the visualised GNP colour profile. This data concludes that the differentiation of glycated protein template from non-glycated protein template based on the GNP-based sensing mechanism is mediated by the low molecular weight AGE products and the same mechanism can be used to distinguish among the proteinaceous and low molecular weight non-proteinaceous glycation products.

The kinetics of the GNP-based colorimetric sensing can be enhanced to greater extends by simply increasing the concentrations of Fruc-Hb and gold salt, thus narrowing down the response time. When the concentrations of the Fruc-Hb and gold salt was increased four times than the concentrations used initially in this study, GNP formation was completed within 1 day, substantiating the use of the proposed colorimetric sensor for point-of-care applications (Additional file 1: Figure S5).

**Fig. 6:**
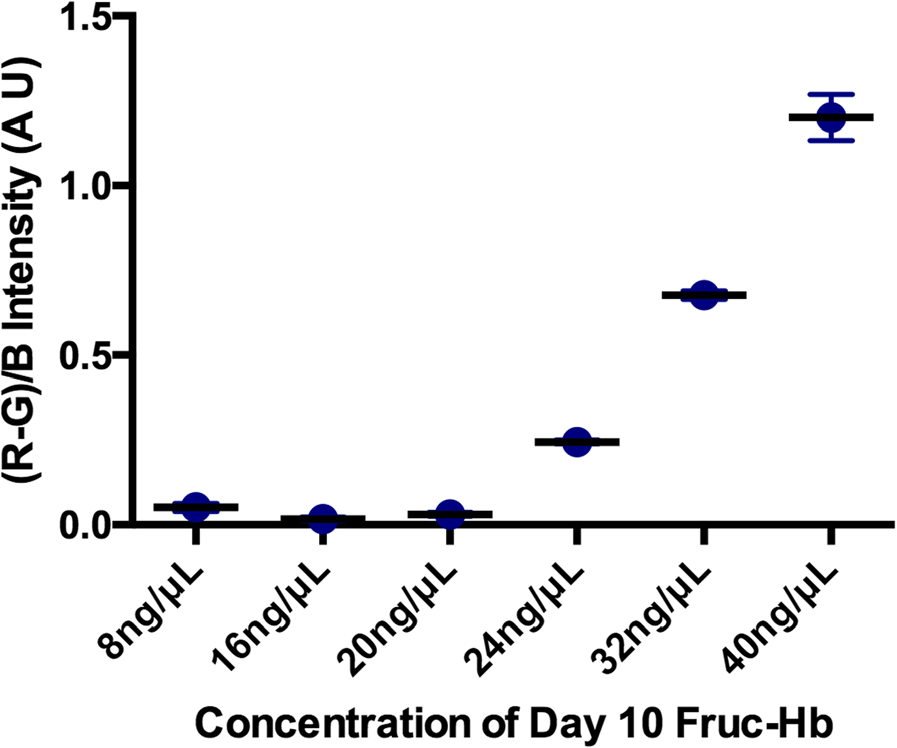
Linearity of detection. The red colour intensity as quantified by (R-G)/B intensity of GNP colloids plotted against the concentration of day 10 Fruc-Hb used for the synthesis. The concentration was varied from 8 to 40 ng/μL

The linearity of detection for this colorimetric sensor was also confirmed using different concentrations of the day 10 glycosylated HbA0 (day 10 Fruc-Hb) (Fig. 6). Colour formation was not prominent enough for the lower concentrations of day 10 Fruc-Hb used, and as the concentration was increased from 20 to 40 ng/μL, the colour intensity increased linearly. This confirms that the colour intensity profile extracted from the GNPs obtained from differentially glycated haemoglobin samples can be used for qualitative measurement of AGEs in respective samples.

## Discussion

The study presented here provides a detailed mechanism of GNP formation from a glycosylated HbA0 template and also discusses how this technique can be expanded for highly sensitive detection of AGE products in a simple manner. Our primary objective for this work was to establish a colorimetric sensing mechanism based on GNPs for the differentiation of glycated and non-glycated samples. The glycated HbA0 was found to be having high reducing property in comparison to the protein and sugar counterparts, enabling formation of stable and mono dispersed GNPs (Fig. 2). Since, among the control samples tested for GNP synthesis, only day 10 Fruc-Hb showed AGE fluorescence, the reactivity can be attributed to the AGE products than a mere structural alterations of the due to glycation (Figs. 1 and 2). To confirm this norm further, the products of glycation obtained from day 10 Fruc-Hb was fractionated to separate the products according to their molecular weights.

Fractionation of glycated Hb segregated two major population of products, high molecular weight proteinaceous (population I) and low molecular weight non-proteinaceous (population II) glycation products (Figs. 3 and 4). When GNPs were synthesised using these fractions of day 10 Fruc-Hb, it was found that only the products belonging to population II, the non-proteinaceous glycation products, were capable of carrying out the synthesis of particles (Fig. 5). This confirmed that the non-proteinaceous AGE products can initiate GNP synthesis by their own and the formation of GNPs from a Fruc-Hb template can be clearly attributed to the AGEs. Also, this opens up the possibility of using this property for the colorimetric detection of AGE products along with the distinction between proteinaceous and non-proteinaceous glycation products of HbA0.

Generally, CARBONYL groups present in the protein and sugar enable the stable synthesis of gold nanostructures in biological syntheses. Here, the generation of AGE products consisting of dicarbonyl functional groups during glycation might be providing the reducing environment for the proposed GNP synthesis [65]. As a matter of fact, the suggested mechanism can be used for the detection of advanced lipid per-oxidation end products (ALEs) as well, since the latter is also characterised by dicarbonyl functional groups and shares structural similarities with AGEs [66]. In short, GNPs synthesised from fractionated glycosylated Hb samples can clearly distinguish between proteinaceous and non-proteinaceous products. Our GNP-based simple colorimetric sensing of glycation products is highly sensitive and can detect nanogram levels of glycation products (S6) in a concentration-dependent manner (Fig. 6). The detection limit using the proposed sensing mechanism for the fractions is 1 ng/μL. The method developed in here can be scaled down to smaller reaction volumes without affecting the reaction kinetics, thus enabling lower sample requirements (data not shown) as well as enhance the reaction kinetics by increasing the concentrations (S5).

## Conclusions

Concisely, in this study, we have demonstrated a colorimetric sensing mechanism for the non-proteinaceous AGE products which is simple and highly sensitive with a detection limit down to nanogram levels. Conditions like type 2 diabetes requires continuous monitoring of sugar levels at regular time intervals. Currently, levels of HbA1c formed as a result of extensive glycation of haemoglobin is used as a diagnostic means for diabetes. Chromatographic [67], electrophoretic and advanced technologies including high-performance liquid chromatography (HPLC) and mass spectrometry (MS) [12, 46–49, 68] are used for HbA1c detection. Early glycation adducts including fructosamines are also indicative of the glycemic control [69] which can be used as a marker for diabetes detection. Monitoring the AGE levels in addition to the HbA1c levels can significantly improve the determination the degree of complexity associated with diabetes, since the advancement of the disease is often associated with AGE formation and is involved in the progression of the disorder as well. Till date, the LC-MS/MS offers the highest selectivity and sensitivity in AGE detection [70]. But when it comes to simple, cost-effective, point-of-care diagnostics, our method can detect few nanograms of the sample compared to other fluorescence emission-based techniques [71, 72]. The method is highly specific for the AGEs, such that sugars and proteins do not develop any colour as such which are the expected interferences in the clinical samples such as blood or serum for the proposed study (Additional file 1 section 7). In this study, we have also segregated the products of glycation to proteinaceous and non-proteinaceous components and proved that non-proteinaceous AGEs are more reactive by the GNP based colorimetric assay. Further research can explore how this idea can be expanded for the distinction between different AGEs and thereby apply it for the diagnosis of organ specific diabetes-related complications.

## Additional File

Additional file 1: An Indian patent has been filed based on this work (Indian patent application number: **201811014098**, dated **April 12, 2018**.) (DOCX 37053 kb)

## Abbreviations

AGEs: Advanced glycation end products
Fruc-Hb: Glycated Hb
GNPs: Gold nanoparticles
HbA0: Haemoglobin A0
SPR: Surface plasmon resonance
