# Supplementary material for: Gold Nanoparticle-Based Detection of Low Molecular Weight AGEs from In Vitro Glycated Haemoglobin A0 Samples
Source: Nanoscale Res Lett. 2018 Dec 4;13:390. doi: 10.1186/s11671-018-2812-y (PMC6277258; doi:10.1186/s11671-018-2812-y)
Supplement: Supplementary file 1 — An Indian patent has been filed based on this work (Indian patent application number: 201811014098, dated April 12, 2018.) (DOCX 37053 kb) [file 11671_2018_2812_MOESM1_ESM.docx]

**Additional file 1**

**S1. Spectroscopic characterisation of Day 0 and Day 10 Fruc-Hb samples**

Fig S1 summarises the formation of AGEs and associated changes in the tertiary and secondary protein structures in the HbA0 samples after 10 days of incubation with fructose *in vitro*. The AGE formation was evaluated by measuring the fluorescence emission at 450nm upon excitation at 350nm (Fig S1A). A gradual increase in the fluorescence intensity with the days of incubation/glycation confirmed the formation of AGE products. We have previously shown that during the course of the fructosylation, the protein backbone undergoes complex structural and configurational changes [56], such as heme loss, unfolding of the protein quaternary and tertiary structure, transition of α-helical structures to beta sheets. Going from Day 0 to Day 10, an increase in the UV absorption at 280nm affirmed the extensive unfolding of the protein structure due to fructose binding accompanied by loss of Heme (complete loss of absorbance at 415nm) (Fig S1B). FRET between tryptophan residues at 340nm (intrinsic protein fluorescence) and AGE structures (Excitation at 350nm and emission at 450nm ) confirmed the formation of AGE in the vicinity of protein backbone (Fig S1C). The CD spectra indicated loss of alpha helix which is associated with the glycation process (Fig S1D). Together, these results confirm the formation of AGEs as the extent of glycation is increased from Day 0 to Day 10 when compared to the non-fructosylated Day 0 Fruc-Hb.
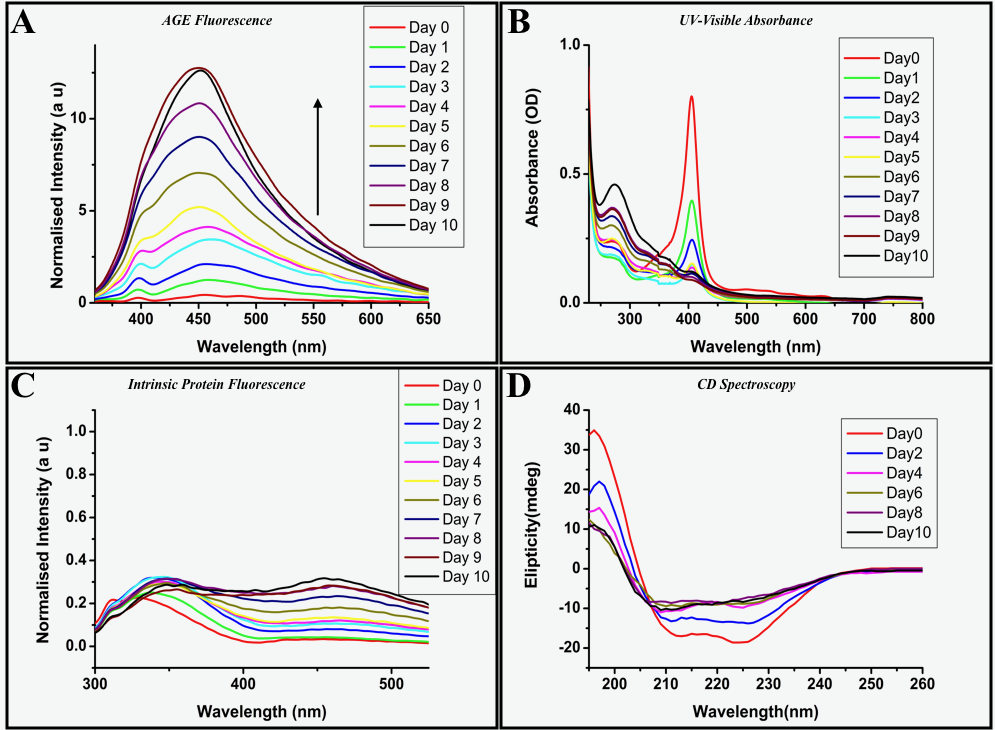


***Figure S1: AGE Fluorescence (A), UV-Vis Spectra (B), Intrinsic protein fluorescence (C) and CD Spectra (D) of glycated HbA0 samples from Day 0 to Day 10 respectively***

**S2. Structural alterations in control Hemoglobin A0 and Fructose after 10 days of incubation**

To confirm if incubation at 37°C induce any significant structural alterations in the protein HbA0 we compared the UV-Visible absorption and Fluorescence emission of Day 10 HbA0 and Day 10 Fruc-Hb. As shown in Fig S2, the incubation as such doesn't cause considerable changes in the protein structure since no heme loss is observed. Whereas, fructosylation leads to complete loss of heme group (Fig S2A ) accompanied by AGE formation (Fig S2B) in Day 10 Fruc-Hb. On the other hand, Fructose shows no measurable differences in the chemical structure after 10 days of incubation (Data not shown).
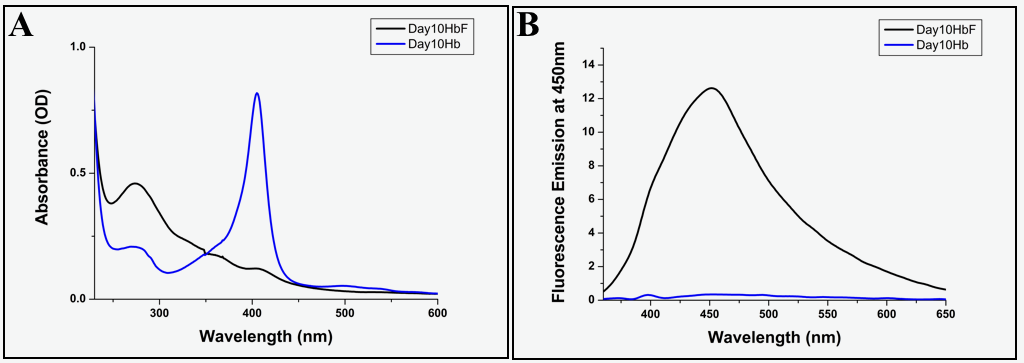


***Figure S2. UV-Visible spectra (A) and Fluorescence emission spectra upon excitation at 350nm (B) of Day 10 HBF and Day 10 HbA0***

**S3. GNP formation from differentially glycated HbA0 samples**

In order to investigate how the GNP formation varies in terms of rate of the reaction, we compared the extinction profiles of GNPs synthesised from Day 1 to Day 10 Fruc-Hb. As shown in Figure S3, as the amount of AGEs for the GNP synthesis is increased (which is proportional to the no. of days of glycation), the SPR intensity of the GNPs increased. An increase in the SPR intensity can be attributed to the presence of higher no. of particles formed which is directly proportional to the amount of AGEs used in the reactant mixture.

***Figure S3. UV-Visible spectra of GNPs synthesised from Day 1 to Day 10 Fruc-Hb samples***

**S4. Structural alterations in control Hemoglobin A0 and Fructose after 10 days of incubation**

SDS Polyacrylamide Gel electrophoresis of Day 0 HbA0, Day 10 HbA0, Day 0 Fruc-Hb and Day 10 Fruc-Hb clearly indicated extensive protein cross linking as a result of glycation in Day 10 fruc-Hb sample. Day 0 HbA0 showed intense bands corresponding to dimers of HbA0. In Day 0 Fruc-Hb sample, the bands have started to fuse and in Day 10 Fruc-HB sample, instead of distinct bands only a smear could be observed confirming cross linking of proteins by the AGEs.
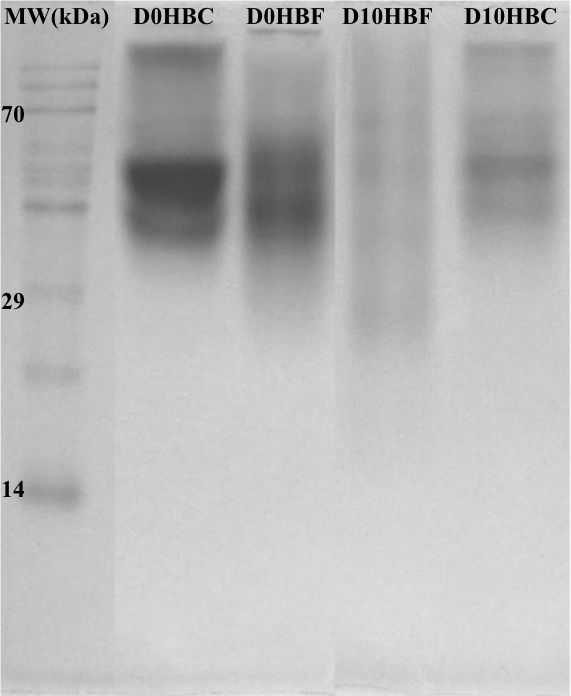


***Figure S4. 12% SDS Polyacrylamide gel electrophoresis of D0HBC, D0HBF, D10HBC, and D10HBF***

**S5. Enhancement of reaction kinetics of the sensing mechanism**

The kinetics of the GNP based colorimetric sensing can be increased by enhancing the concentrations of the reactions, that is Gold salt and Fruc-Hb. When concentrations of the reactants was increased, the response time was found to be decreased by 4 times. Fig S4 represents the spectroscopic characterisation of the GNPs synthesised from Day 10 Fruc-Hb (A) with lower concentration of the reactants and (B) higher concentration of the reactants. As evident from the figure, changing the concentrations only alter the kinetics and response time of the reaction and not the nature of the final products.
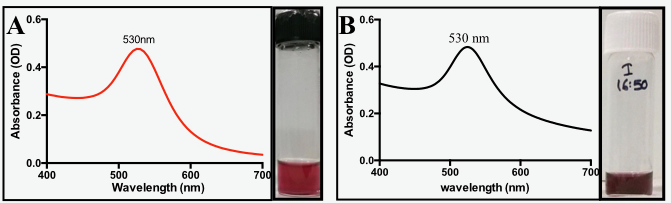


***Figure S5. (A) Visible absorption spectra of Day 10 HBF_GNPs _4 days (lower concentration of the substrates ) and Day 10 HBF_GNPs_1 Day with (higher concentration of the substrates )***

**S6. Detailed calculation of final concentrations of Day 10 Fruc-Hb and the Fractions**

***Day 10 Fruc-Hb concentration in the GNP synthesis***

Concentration of Day 10 Fruc-Hb - 1mg/mL

Volume of Day 10 Fruc-Hb taken for GNP synthesis - 50 μL in 4 mL

Final concentration of Day 10 Fruc-Hb required to synthesise GNPs - 50μg in 4mL/ 12.5μg in 1 mL/ 12.5**ng/μL**

***Concentration of fractions from Day 10 Fruc-Hb in the GNP synthesis(without citrate)***

Amount of Day 10 Fruc-Hb loaded into the G25 column - 600ug (600μL of 1mg/1mL)

Total volume of the column - 15mL

Volume of one single fraction - 500μL

Approximate concentration of the protein/AGE products after eluting through the column - 600μg/15mL= 40μg/mL = **4μg/100μL**

Volume of fractions taken for GNP synthesis - 100μL in 4mL (4μg in 4mL)

Final concentration of the fractions required to synthesise GNPs - 4μg in 4ml / 1μg in 1mL / **1ng in 1μL**

**S7. Detection of Interferences in the colorimetric sensing of AGEs by Human Serum Albumin (HSA)
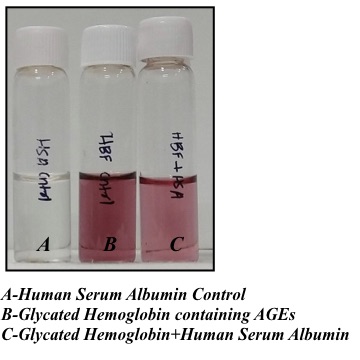
**

***Figure S6. (A) GNP synthesis in presence and absence of the serum albumin with only Human serum albumin as a control. Photographs of colloidal solutions of GNPs synthesised using A. Human Serum Albumin B. AGEs C. AGEs in the presence of Human Serum Albumin***

Serum Albumin is the most abundant protein in serum. Her we have used this as a control to study any interferences of it during the GNP synthesis using AGEs. The results for GNP synthesis from AGEs in the absence and presence of the Human Serum Albumin are presented in figure S6. The concentration of Albumin was kept similar to that of Hemoglobin that was used for glycation (Methods: Glycation of HbA0). The appearance of colour in the AGE containing samples were kinetically faster, when the results were observed upto 16 hours. As evident from the figure, Human Serum Albumin (HSA) by itself didn’t contribute to GNP synthesis and doesn’t produce any significant interferences in the colorimetric sensing of AGEs even after the long incubation.
